# Clinical performance of uncoated and precoated polymer mesh base ceramic brackets

**DOI:** 10.1186/s40510-018-0253-x

**Published:** 2019-01-28

**Authors:** Hüdanur Yılmaz (née Huda Abulkbash), Selma Elekdag-Türk

**Affiliations:** 1 İlkadım/Samsun, Turkey; 2 Department of Orthodontics, Faculty of Dentistry, University of Ondokuz Mayis, Atakum/Samsun, 55139 Turkey

**Keywords:** Operator-coated InVu ceramic brackets, Readi-Base eXact pre-applied adhesive InVu ceramic brackets, Bracket failure, Tie-wing fractures, Modified remnant index

## Abstract

**Background:**

To evaluate the clinical performance of the two types of InVu ceramic brackets. The clinical performance of these brackets was measured by determining failure as well as survival rates and tie-wing fractures. Enamel surface evaluation following bracket and remnant removal was performed.

**Subjects and methods:**

Forty non-extraction patients (31 females, 9 males) with a mean chronological age of 16 years 4 months composed this study. Bonding was performed with a split-mouth design using operator-coated and Readi-Base eXact InVu brackets. During the treatment period (45.89 ± 2.0 weeks), the failed brackets were recorded as well as the brackets with tie-wing fractures. Debracketing was undertaken with a ligature cutter (delamination technique) as recommended by the manufacturer. A modified remnant index (MRI) was used to visually evaluate the amount of remnants remaining on the tooth surface. Horizontal crack evaluation was carried out via transillumination.

**Results:**

Operator-coated InVu brackets demonstrated a bond failure rate of 2.6%. This value was 6.8% for the Readi-Base eXact InVu brackets. Failure rates as well as survival rates presented a statistically significant difference (*P* = 0.006). A higher bond failure for the premolar teeth when compared to incisor teeth, as well as a higher bond failure in the lower arch when compared to the upper arch was found. These findings were statistically significant (*P* = 0.000 and *P* = 0.007, respectively). The effect of gender on bond failure rate (*P* = 0.508) and survival rate (*P* = 0.503) was not statistically significant. Both bracket types showed comparable results for tie-wing fractures (*P* = 0.174). A statistically significant difference was obtained for the MRI scores (*P* = 0.000). No horizontal enamel cracks were observed for both bracket types.

**Conclusion:**

The operator-coated InVu brackets demonstrated a lower failure rate when compared to the Readi-Base eXact pre-applied adhesive InVu brackets. The debonding procedure was safe for both bracket types.

## Introduction

The success of fixed appliance therapy depends on orthodontic attachments having reliable bond strengths and bond failure rates as low as possible.

The median bond failure rate was reported to be approximately 5% in a survey. This survey also reported an increase in the use of ceramic as well as adhesive precoated brackets (APC) [1].

In 1991, APC brackets were introduced. This type of bracket is manufactured with a uniform coating of adhesive covering the entire bracket base, thereby simplifying the bonding procedure [2].

Upon a literature search, three clinical trials comparing the APC system with the conventional approach, i.e., the operator-coating of Transbond XT light-cure adhesive, were encountered. These studies had a split-mouth design, and no significant difference concerning the bond failure between the two approaches was obtained [3–5]. One of these studies was carried out with ceramic brackets [5].

Kula et al. [3] concluded that both approaches were equally effective. It was pointed out that the stainless steel brackets were placed by relatively inexperienced operators. A bond failure of 7.5% for an observation period of 12 months was reported.

The results of Wong and Power [4] also showed that both approaches were equally effective. Here, the stainless steel brackets were placed by one operator. An overall bond failure rate of 7.37% was reported. Each subject was monitored for 6 months.

The study by Verstrynge et al. [5] reported no bond failures for both approaches. This study was performed with polycrystalline, true-twin ceramic brackets with a micro-mechanical retention mechanism. During treatment, only one tie-wing fractured in the APC group. The authors concluded that this tie-wing fracture was most likely due to tieing a metal ligature too strongly.

In August 2013, InVu ceramic brackets (TP Orthodontics Inc., La Porte, IN, USA) with Readi-Base eXact pre-applied adhesive were introduced. The adhesive application is in an X-shape on the bracket base. The manufacturer claims that superior bond strength with minimal flash clean up around the bracket base is made possible with this X-shape adhesive design [6].

The InVu bracket is an injection-molded polycrystalline ceramic bracket incorporating a flexible polymer mesh base. The manufacturer states that the InVu brackets ensure a safe debracketing process [7].

To date, no in vivo studies assessing the debracketing characteristics of the InVu ceramic bracket exist; however, three in vitro studies are present [8–10]. These in vitro studies concluded that the InVu bracket preserves the original enamel surface after debracketing.

The specific objectives of this study were as follows:

1. To determine bond failures and survival rates of the InVu brackets (operator-coated InVu versus precoated InVu brackets).
2. To record the number of tie-wing fractures.
3. To record the amount of the remnants (i.e., adhesive and polymer mesh base) left on the enamel surface after debracketing.
4. To evaluate the integrity of the enamel surface following remnant removal.

## Subjects and methods

Ethical approval for this clinical study evaluating bracket failure (bond failure as well as tie-wing fracture) and debonding characteristics of pre-applied light cure adhesive-polymer mesh base ceramic brackets (Readi-Base eXact ceramic brackets, TP Orthodontics, Inc., La Porte, IN, USA) versus uncoated polymer mesh base ceramic brackets (InVu ceramic brackets, TP Orthodontics, Inc.) was obtained from the Research Ethics Committee at the University of Ondokuz Mayis. This study was carried out in the Department of Orthodontics, Faculty of Dentistry, University of Ondokuz Mayis. One operator was responsible for the treatment of the patients, recording of bond failures and recording of tie-wing fractures, remnant, and crack evaluations.

**Fig. 1:**
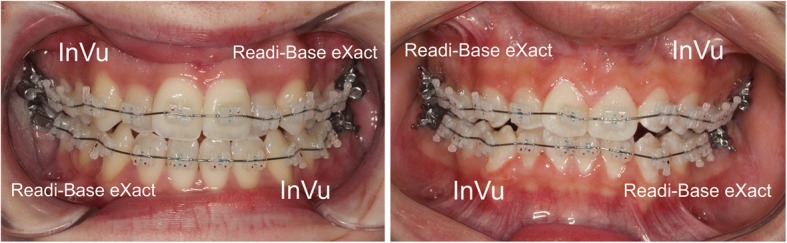
Intervention A and intervention B

The sample size was estimated by G*Power (version 3.1.9.2) [11] according to a previous study [12] on bracket failure rate (80% power, 5% significance level; two-tailed). To observe a 1.0% difference for the failure rate, a minimum sample size of 269 brackets for each intervention was required for the detection of a significant difference between intervention A and intervention B (Fig. 1). The sample size was increased to 40 patients (400 brackets for each intervention) to account for possible drop-outs.

The eligibility criteria for the patients were as follows:

- Non-extraction patients with a class I molar relationship.
- Absence of severe rotations. Severe rotations would have prevented bracket placement or correct bracket placement at first appointment.
- A normal overbite value.
- Presence of all teeth with intact buccal enamel.
- Absence of pretreatment of teeth with any chemical agents.
- Absence of horizontal enamel cracks.

The presence of horizontal enamel cracks was identified with the help of a portable dental transilluminator (DiscoverLight LED, Burstein, Enterprises Inc., Canada).

Due to unexpected family relocation, the number of participants dropped down to 38 (1 patient from each intervention) shortly after the beginning of this study. The details of the sample size, mean age, and patient distribution by gender, age, and tooth type are presented in Table 1.

**Table 1:** Sample characteristics

	Number	Percentage
Number of total patients	38	–
Distribution of patients by gender		
Female	30	78.94
Male	8	21.05
Distribution of patients by age		
12–13	6	15.80
14–15	12	31.60
16–18	9	23.70
> 18	11	28.90
Mean age		
16 years and 4 months		
Number of brackets	760	–
Distribution of brackets by adhesive type		
Operator-coated InVu	380	50.00
Readi-Base eXact InVu	380	50.00
Distribution of brackets by gender		
Female	580	76.32
Male	180	23.68
Distribution of brackets by dental arch		
Upper arch	380	50.00
Lower arch	380	50.00
Distribution of brackets by tooth type		
Incisor	300	39.50
Canine	152	21.00
Premolar	300	39.50

Before the beginning of treatment study models, panoramic X-rays, photographs, and consent forms signed by patients and parents (if necessary) were obtained.

The 40 patients were randomly distributed into intervention A and intervention B (Fig. 1) with a computer-generated list.

Prior to the bonding procedure, all teeth were polished with a fluoride-free pumice with rubber cups. The teeth were etched with 37% phosphoric etchant liquid gel (3 M Unitek, Monrovia, Cal) for 30 s, rinsed, and dried until the buccal surfaces of the etched teeth appeared chalky white in color. After etching, a thin coat of Transbond XT primer (3 M Unitek) was applied onto the etched enamel. The Readi-Base eXact InVu brackets were pressed onto the appropriate tooth. Excess adhesive flash (EAF) was removed with an explorer. Light-curing was performed directly through the labial surface for 30 s as recommended by the manufacturer. A brand new, visible light-curing unit (Led.G, Guilin Woodpecker Medical Instrument Co., Ltd., Guangxi, China) with an output power of 850–1000 milliwatts/square centimeter (mW/cm^2)^ was used.

The InVu brackets were bonded with Transbond XT adhesive (3 M Unitek) in the contralateral quadrants. EAF was removed, and light-curing was performed. Each adhesive was highly filled according to the companies.

A thin layer of cement (Transbond Plus Light Cure Band Adhesive, 3 M Unitek) was placed on the occlusal surfaces of the lower first molars to hinder potential occlusal interferences.

Treatment was started with 0.014 in. HANT (heat-activated nickel titanium) archwires. The 0.022-in. slot McLaughlin Bennett Trevisi (MBT) prescription was used. Fixed orthodontic appliance care was explained to patients and parents (if necessary). The patients were instructed to check for loose brackets or bracket fractures on a daily basis. If bond failure or a tie-wing fracture occurred, they were asked to immediately contact and visit the orthodontist.

The course of active orthodontic treatment ranged between 10 and 12 months. Only elastic ligatures were used. The patients were controlled every 4 weeks. Brackets with bond failures and brackets with tie-wing fractures were replaced with metal brackets. Patients were informed about this procedure before the beginning of treatment.

At the end of treatment the ceramic brackets were debonded according to the manufacturer’s instructions. After placing a sharp ligature cutter as close as possible to the enamel surface, the ligature cutter was squeezed mesio-distally (delamination technique). The archwire was left ligated during the debonding procedure to prevent the “popping-off” of the brackets. The ligature cutters were sharpened for every patient, as the plier blades lose their sharpness.

The amount of remnants remaining on the tooth were visually evaluated according to a modification of the original adhesive remnant index (ARI) [13]. This modified index, the modified remnant index (MRI), had the following scores: 0, no adhesive left on the tooth; 1, less than half of the adhesive left on the tooth; 2, more than half of the adhesive left on the tooth; 3, all adhesive left on the tooth, with a distinct impression of the bracket mesh base; 4, all adhesive including part of the polymer mesh base left on the tooth; 5, all adhesive including all of the polymer mesh base left on the tooth.

The remnants were removed from the enamel surface with a 12-fluted tapered tungsten carbide bur (Hager and Meisinger GmbH Neuss, Germany) in a slow speed handpiece with a speed of 25,000 rpm. From time to time a water spray was used to prevent dust formation. Each tooth was then reevaluated for horizontal cracks with the portable dental transilluminator (DiscoverLight LED, Burstein, Enterprises Inc., Canada).

At the completion of treatment, orthodontic study models, panoramic X-rays, intra-oral, and extra-oral photographs were taken. The retention appliances were placed.

### Statistical analyses

Bond failure rates were determined for each bracket, dental arch, type of tooth (incisor, canine, and premolar), and patients’ gender. The chi-square test was used to compare the failure rates (*P* < 0.05).

The survival rates of the brackets were estimated with the Kaplan-Meier test. Bracket survival distributions with respect to bracket, dental arch, and type of tooth (incisor, canine, and premolar) as well as patients’ gender were compared using the log-rank test (*P* < 0.05).

The differences in MRI scores between the failed brackets during treatment were determined with the chi-square analysis (*P* < 0.05). Likewise, the chi-square analysis was used to determine the significant differences for the MRI scores between the brackets after the debonding procedure (*P* < 0.05).

Furthermore, the chi-square analysis was used to find out the significant differences of the wing fractures between the brackets (*P* < 0.05).

Horizontal cracks could not be subjected to a statistical analysis, since no horizontal cracks were detected at the end of treatment, i.e., following debonding.

## Results

**Fig. 2:**
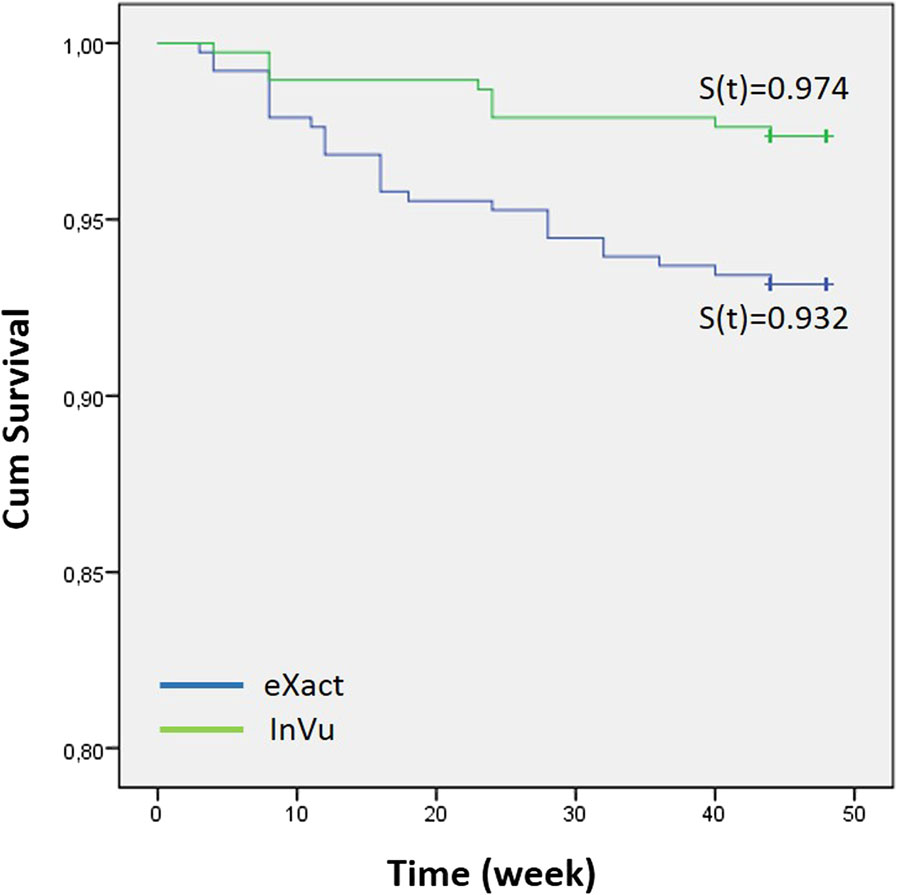
Bracket survival distribution

During the observation period (45.89 ± 2.0 weeks), a total of 36 brackets failed: 10 (2.6%) for the InVu brackets and 26 (6.8%) for the Readi-Base eXact InVu brackets (Table 2). A significant difference between the failure rates (*χ*^2^ = 7.465; *P* = 0.006) was obtained. The bracket survival curves were plotted with the Kaplan-Meier estimate for the observation period (Fig. 2).

**Table 2:** Bond failure rates

InVu			eXact			*P*	Log-rank test
No failure	Failure	Failure rate	No failure	Failure	Failure rate		
370	10	2.6%	354	26	6.8%	0.006*	0.006

*χ^2^ = 7.465 on 1 df

The bracket type demonstrated a significant influence on the bracket survival rates (*P* = 0.006). The probabilities of having bonded brackets still in place at the end of the observation period were 0.974 and 0.932 for the InVu and Readi-Base eXact InVu brackets, respectively (Fig. 2).

Bond failure rates were 1.0% (3 brackets) for incisor teeth and 10.9% (33 brackets) for premolar teeth (Table 3). Significant differences were observed for the failure rates of incisor and premolar teeth (Table 3, *P* = 0.000).

**Table 3:** Bond failure rates for type of tooth (incisor, canine, and premolar)*

	No failure	Failure	Failure rate (%)	Log-rank test
Incisor	301	3	1.0	0.000
Canine	152	–	0.0	
Premolar	271	33	10.9	

*χ^2^ = 42.251 on 2 df; *P* = 0.000

**Fig. 3:**
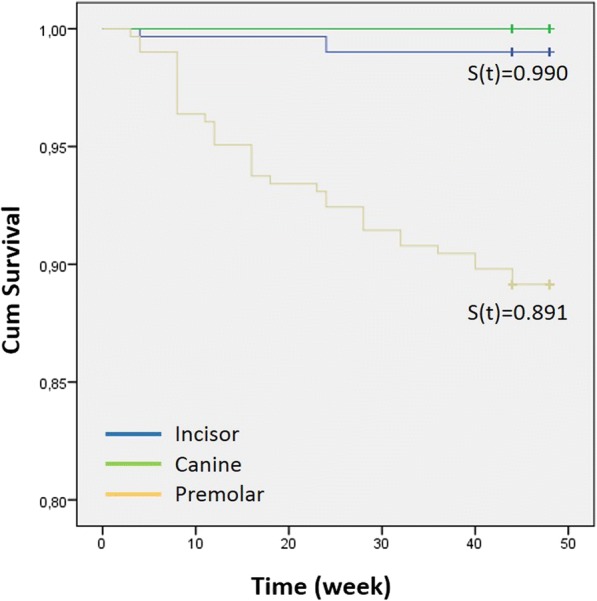
Bracket survival distribution for tooth type (incisor, canine, and premolar)

Figure 3 shows the influence of arch location on the bracket survival rate. The log-rank test showed significant differences between the incisor and premolar teeth in terms of survival rate (*P* = 0.000).

Bond failure rates were 2.6% (10 brackets) and 6.8% (26 brackets) in the upper and lower arches, respectively. This difference was statistically significant (Table 4; *P* = 0.007).

**Table 4:** Bond failure rates for upper and lower dental arches*

	No failure	Failure	Failure rate (%)	Log-rank test
Upper	370	10	2.6	0.007
Lower	354	26	6.8	

*χ^2^ = 7.465 on 1 df; *P* = 0.006

**Fig. 4:**
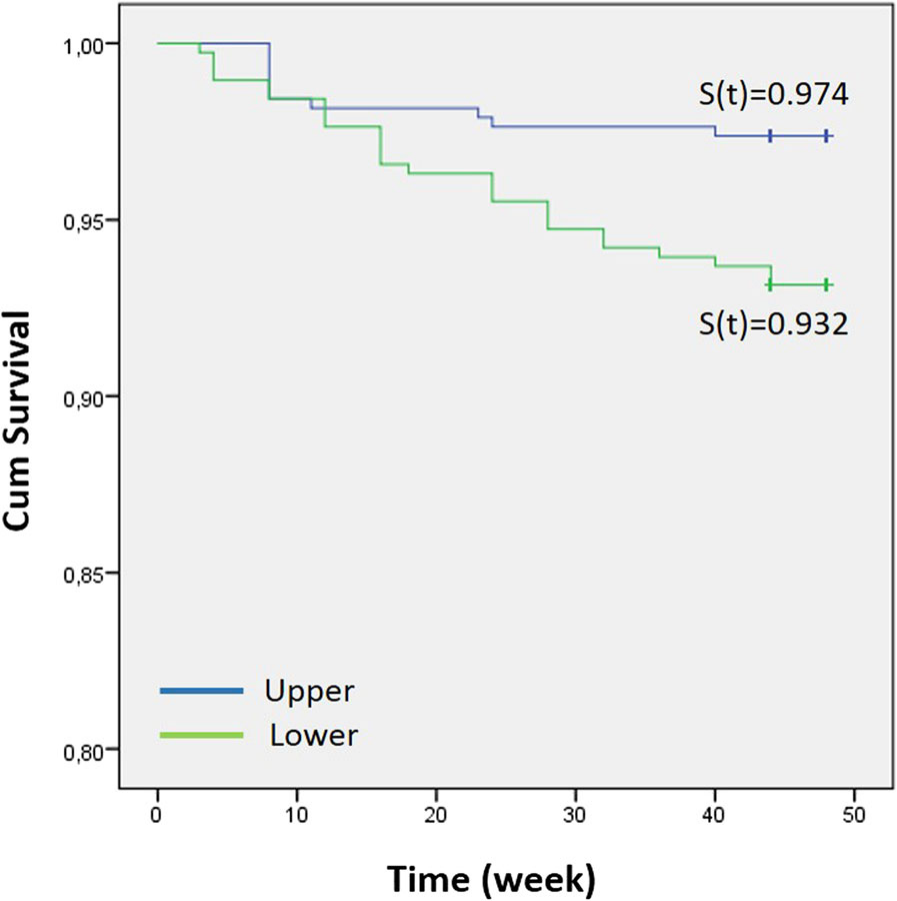
Bracket survival distribution for dental arches

The influence of the dental arches on bracket survival rate is shown in Fig. 4.

The log-rank test showed a significant difference between upper (*S*[*t*] = 0.974) and lower (*S*[*t*] = 0.932) dental arches (*P* = 0.007).

Female and male patients presented a 5.0% (30 brackets) and 3.8% (6 brackets) failure rate, respectively. This difference was not statistically significant (Table 5; *P* = 0.508).

**Table 5:** Bond failure rates for female and male patients*

	No failure	Failure	Failure rate (%)	Log-rank test
Females	570	30	5.6	0.503
Males	154	6	3.8	

*χ^2^ = 0.437 on 1 df; *P* = 0.508

**Fig. 5:**
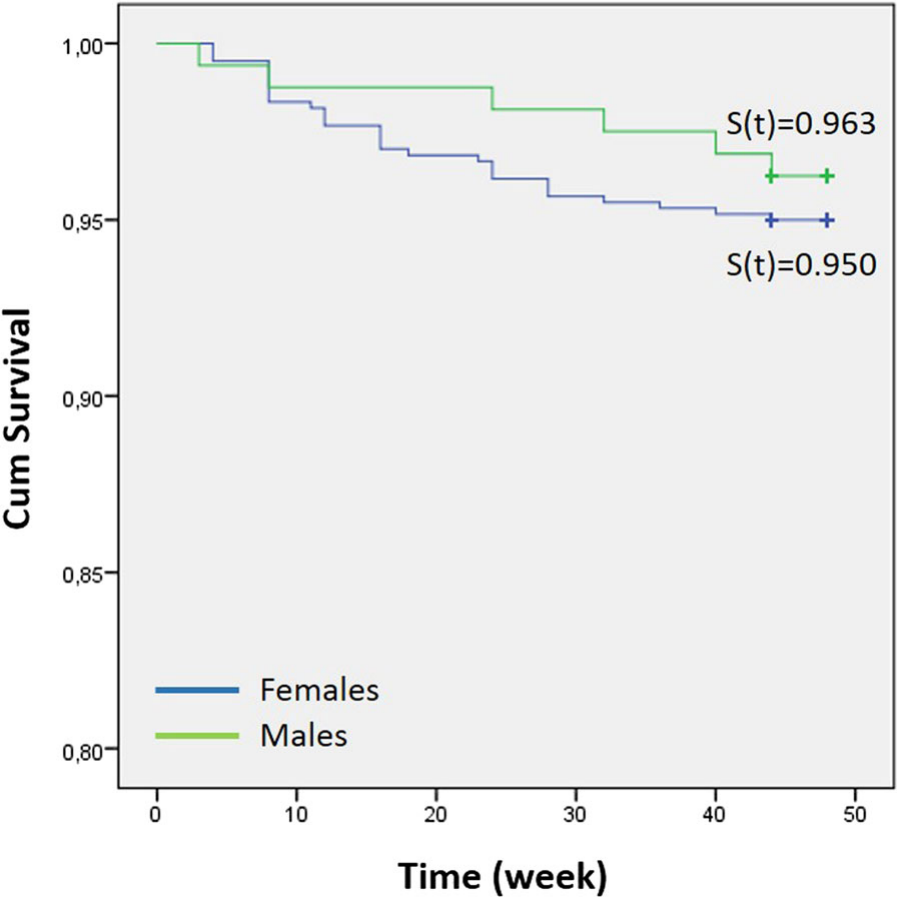
Bracket survival distribution for female and male patients

The influence of gender on the bracket survival rate is shown in Fig. 5. No significant difference between females (*S*[*t*] = 0.950) and males (*S*[*t*] = 0.963) was observed with the log-rank test (*P* = 0.503).

Frequency distribution and the result of the *χ*^2^ analysis of the tie-wing fractures are presented in Table 6. No significant difference was observed between the two bracket types (*P* = 0.174).

**Table 6:** Frequency distribution and the result of the *χ*^2^ analysis of the wing fracture*

	No fracture	Fracture	Fracture rate (%)
eXact	367	13	3.4
InVu	373	7	1.8

*χ^2^ = 1.849 on 1 df; *P* = 0.174

After debonding procedures, frequency distribution and the result of the *χ*^2^ analysis of the MRI scores are presented in Table 7. Significant difference was observed between the brackets (*P* = 0.000).

**Table 7:** Frequency distribution and the result of the *χ*^2^ analysis of the modified remnant index (MRI) after debonding procedures*

	MRI scores						
	0	1	2	3	4	5	Total
eXact	121	105	26	4	25	60	341
InVu	133	55	10	18	34	113	363

*χ^2^ = 49.183 on 5 df; *P* = 0.000

## Discussion

The precoated InVu brackets failed at a higher rate than the operator-coated InVu brackets. At the end of treatment (45.89 ± 2.0 weeks), the bond failure rates were 6.8% (26 brackets) for the precoated brackets (Readi-Base eXact) and 2.6% (10 brackets) for the operator-coated brackets. The failure rates for both approaches presented a statistically significant difference.

These results do not agree with the results of Kula et al. [3], Wong and Power [4], and Verstrynge et al. [5], who reported that both approaches proved to be equally effective. This outcome might be due to the distinctive X-shaped adhesive application on the bracket base. When this type of bracket is placed and pressed onto the tooth, the X-shaped adhesive material may not homogenously spread over the entire bracket base and the enamel. This might result in gap formation at the tooth-adhesive-bracket complex, weakening the bonds. Subsequently, the seeping and leaking of oral fluids and microorganisms into these gaps may further undermine these bonds [14, 15]. Matasa [16] stated that adhesives are “eaten away” by microorganisms and concluded that this occurrence may possibly be a culprit for bond failure.

The operator had no previous experience with precoated brackets. Yet, the bonding and handling procedures of these brackets were strictly performed according to the guidelines of the manufacturer [6].

Bracket failure rates are an accepted method for the evaluation of bracket performance, allowing the comparison with the results of other studies [17]. In addition to the simple event of failure, survival rate assessment allows consideration of the time interval before bracket failure. Hence, survival rate application allows certain important differences to be underlined, which is not possible with failure rates [17].

In the present study, the survival rates were 0.974 and 0.932 for the uncoated InVu and for the precoated InVu brackets, respectively. The bracket type demonstrated a significant influence on the bracket survival rate. The obtained survival rates indicate that the probability of having bonded brackets still in place at the completion of treatment was 97 and 93% for the uncoated and for the precoated brackets, respectively.

During the course of the present study (45.89 ± 2.0 weeks), bond failures were encountered with 3 incisor (1.0%) and 33 premolar brackets (10.9%). A significant failure rate difference between anterior and posterior regions was present. Numerous publications in agreement with these results exist [3, 18–22]. Interestingly, two studies reported the complete opposite, namely a higher bracket failure rate in the anterior region [23, 24]. Pettemerides et al. [23] presented no explanations for this result, whereas Manning et al. [24] stated that habits such as nail biting and pen chewing might be responsible.

The reasons for the higher bracket failure in the posterior region of the dentition were summarized as follows: increased difficulty in moisture control of the posterior segments of the dentition [3, 18–22]; greater occlusal stresses and masticatory forces applied to the posterior teeth [3, 18, 19, 21, 22]; inadequate adaptation of the bracket base due to the buccal anatomy of the premolars [3, 18]; individual variation, such as hypersalivation [18]; access and visualization difficulties during bonding [3, 22]; and the greater extent of aprismatic enamel on posterior teeth [20, 22]. Aprismatic enamel is considered to result in an “inferior” etch pattern [25].

All of these reasons, except hypersalivation, may have led to the pronounced bracket failure of the posterior teeth in the present study. In fact, the bracket failure of the posterior teeth (premolars) was 11 times higher than for the incisor teeth. The distinct X-shape adhesive application of the precoated brackets might have exacerbated this result. Furthermore, effective marginal sealing at the tooth-adhesive-bracket complex might not have been able to occur due to the buccal anatomy of the premolars, thereby further weakening the bond.

The bracket survival rate showed a significant difference between the anterior and posterior teeth. The shape of the survival “curve” for the premolars demonstrated a steep staircase appearance when compared to the survival curves’ of the canines and incisors. The survival rates indicated that the probability of having bonded brackets still in place was 100, 99, and 89% for the canines, incisors, and premolars, respectively.

Miles et al. [26] reported that the distribution of bracket failures between the upper and lower dental arches has demonstrated some variation; however, the trend most often observed is a higher risk of bond failure in the mandibular dental arch. The two main reasons presented for this trend are occlusal interferences between the brackets of the lower and the upper dental arch during the first phase of orthodontic treatment and the higher masticatory forces on the mandibular bonds [21, 22].

In the present study the bond failure rate was 2.6% (10 brackets) in the upper arch and 6.8% (26 brackets) in the lower arch. This presented a statistically significant difference. To circumvent the problem of occlusal interferences between the brackets of the upper and the lower dental arches, a thin layer of cement was placed on the occlusal surfaces of the lower first molars. Thus, the higher masticatory forces on the mandibular bonds most likely caused the higher number of failed mandibular bonds (brackets). Also, the difficulties with moisture control during bonding of the mandibular posterior teeth might have contributed to this outcome [21].

The bracket survival rate demonstrated a significant difference between upper and lower dental arches. The obtained survival rates indicated that the probability of having bonded brackets still in place at the end of the observation period for the upper and lower dental arches was 97 and 93%, respectively.

Starnbach and Kaplan [27] concluded that gender is an important predictor for compliance. According to these researchers, females comply better than males. They ascribed this to the fact that orthodontic treatment is dealing with the improvement of esthetics, which is of greater concern to girls than boys. Furthermore, these researchers stated that girls tend to mature earlier than boys and, therefore, may adopt a more adult attitude towards orthodontic treatment.

In light of this information, one might expect higher bond failure rates for boys. In fact, Adolfsson et al. [21], Koupis et al. [22], and Manning et al. [24] noted higher bond failure rates for male patients. Adolfsson et al. [21] speculated that girls are more careful with their appliances than boys. Surprisingly, although not statistically significant, Cal-Neto et al. [28] received a higher bond failure rate for female subjects (7.23%) when compared to male subjects (3.70%). The results of the present study are in agreement with the study of Cal-Neto et al. [28]. The bond failure rates were 5.6 and 3.8% in female and male patients, respectively. Nevertheless, the bond failure rates as well as the bracket survival rates did not demonstrate a statistically significant difference.

The variability in bond failure rate is interesting. The comparison between clinical studies evaluating bond failure, even with identical materials and with operators having a similar background, has to be approached with caution. Numerous factors, such as the socioeconomic and dental status of patients, malocclusion classification and the resultant mechanotherapy, masticatory forces varying with facial type, and culturally influenced dietary habits may all have an influence on the failure rates [29].

Ceramic bracket tie-wing fractures constitute a serious problem, since the effective ligation of the archwire to the impaired bracket is no longer possible. Furthermore, impaired brackets are prone to complete fracture. Thus, as a risk management procedure, the debonding of the impaired bracket and its replacement with a new bracket is required.

In the present study, 13 (3.4%) and 7 (1.8%) brackets had to be renewed for the precoated and operator-coated brackets, respectively. Even though the outcome was not statistically significant, almost twice as many tie-wing fractures occurred for the precoated brackets. The reason for this is unclear, since both were polycrystalline, true-twin brackets. Nevertheless, one might speculate that the operator-coated adhesive imparts an enhanced, uniform cross-stabilizing effect when compared to the X-shaped adhesive, thereby making the operator-coated bracket more robust.

Tie-wing fractures (failures) as well as bond failures necessitate rebonding. This translates into a combined failure rate of 10.2% (6.8% + 3.4%) and 4.4% (2.6% + 1.8%) for precoated and operator-coated brackets, respectively. The outcome for the operator-coated bracket (4.4%) is encouraging, when compared to the failure rate data presented by Keim et al. [1], namely 5%.

The InVu bracket is an injection-molded polycrystalline bracket with a thin flexible polymer mesh base. Three in vitro studies [8–10] concluded that the InVu bracket preserves the enamel surface after debonding. Nevertheless, bracket base fractures at the ceramic/polymer interface were noted [8, 9]. The results of the present clinical study are in agreement with these results.

In the present study the bond failure sites were characterized with the MRI ranging from 1 to 5. The higher scores indicate fractures that are closer to the adhesive-polymer mesh base interfaces. Remnants left on the enamel surface are considered as an advantage by some researchers, since this type of outcome protects the enamel surface [30]. In the present study, a statistical significant difference was obtained for the MRI scores. Operator-coated brackets had almost twice as many MRI scores of 5 (all adhesive including all of the polymer mesh base left on the tooth) when compared to the precoated brackets, i.e., 113 versus 60 teeth. This might be due to a stronger bond between the enamel-adhesive flexible polymer mesh base complex for these 113 teeth.

On the other hand, 133 operator-coated InVu teeth and 121 Readi-Base eXact teeth had an MRI score of 0 (no adhesive left on the tooth). This location is considered disadvantageous [30]. Nevertheless, the enamel surface was intact with no horizontal cracks as determined via transillumination for all teeth with both types of brackets. The flexible polymer mesh base provides a protective barrier, an area for stress absorption, between the ceramic part of the bracket and the enamel. Furthermore, the absence of horizontal cracks is an indication of a sound debonding technique [18].

It should be pointed out that MRI scores of 4 and 5 are indicative of fractures at the ceramic/polymer mesh base interface. The polymer mesh base remnants were easily removed with the same bur used to remove the adhesive remnants. Furthermore, no fracturing of the ceramic part for any of the brackets was encountered.

## Conclusions

The operator-coated InVu brackets demonstrated a lower failure rate when compared to the precoated InVu brackets. The debonding procedure was safe for both bracket types. This is of utmost importance in an increasingly litigious society.

## Abbreviations

APC: Adhesive precoated
ARI: Adhesive remnant index
EAF: Excess adhesive flash
HANT: Heat-activated nickel titanium
MBT: McLaughlin Bennett Trevisi
MRI: Modified remnant index
mW/cm^2^: Milliwatts/square centimeter
